# Adherence to EAT-Lancet dietary recommendations for health and sustainability in the Gambia

**DOI:** 10.1088/1748-9326/ac9326

**Published:** 2022-10-06

**Authors:** Zakari Ali, Pauline F D Scheelbeek, Jyoti Felix, Bakary Jallow, Amanda Palazzo, Alcade C Segnon, Petr Havlík, Andrew M Prentice, Rosemary Green

**Affiliations:** 1 Nutrition and Planetary Health Theme, MRC Unit The Gambia at the London School of Hygiene and Tropical Medicine, Banjul, The Gambia; 2 Faculty of Epidemiology and Population Health, London School of Hygiene and Tropical Medicine, London, United Kingdom; 3 Centre on Climate Change and Planetary Health, London School of Hygiene and Tropical Medicine, London, United Kingdom; 4 National Nutrition Agency (NaNA), Banjul, The Gambia; 5 International Institute for Applied Systems Analysis, Laxenburg, Austria; 6 CGIAR Research Program on Climate Change, Agriculture and Food Security (CCAFS), International Crops Research Institute for the Semi-Arid Tropics (ICRISAT), Bamako, Mali; 7 Alliance of Bioversity International and International Center for Tropical Agriculture (CIAT), Dakar, Senegal; 8 Faculty of Agronomic Sciences, University of Abomey-Calavi, Cotonou, Benin

**Keywords:** dietary sustainability, EAT-Lancet diet, diet composition, Gambia, environmental footprint

## Abstract

Facilitating dietary change is pivotal to improving population health, increasing food system resilience, and minimizing adverse impacts on the environment, but assessment of the current ‘status-quo’ and identification of bottlenecks for improvement has been lacking to date. We assessed deviation of the Gambian diet from the EAT-Lancet guidelines for healthy and sustainable diets and identified leverage points to improve nutritional and planetary health. We analysed the 2015/16 Gambian Integrated Household Survey dataset comprising food consumption data from 12 713 households. Consumption of different food groups was compared against the EAT-Lancet reference diet targets to assess deviation from the guidelines. We computed a ‘sustainable and healthy diet index (SHDI)’ based on deviation of different food groups from the EAT-Lancet recommendations and modelled the socio-economic and geographic determinants of households that achieved higher scores on this index, using multivariable mixed effects regression. The average Gambian diet had very low adherence to EAT-Lancet recommendations. The diet was dominated by refined grains and added sugars which exceeded the recommendations. SHDI scores for nutritionally important food groups such as fruits, vegetables, nuts, dairy, poultry, and beef and lamb were low. Household characteristics associated with higher SHDI scores included: being a female-headed household, having a relatively small household size, having a schooled head of the household, having a high wealth index, and residing in an urban settlement. Furthermore, diets reported in the dry season and households with high crop production diversity showed increased adherence to the targets. While average Gambian diets include lower amounts of food groups with harmful environmental footprint, they are also inadequate in healthy food groups and are high in sugar. There are opportunities to improve diets without increasing their environmental footprint by focusing on the substitution of refined grains by wholegrains, reducing sugar and increasing fruit and vegetables consumption.

## 1. Introduction

Globally, over 40% of all men and women are overweight or obese, 195 million children under five years suffer from stunted growth or wasting [1] and micronutrient deficiencies affect over 2 billion people [2]. Furthermore, over the last decade, slow progress has been made in meeting global targets on maternal and child nutrition; and no country is currently on course to meet global diet-related non-communicable disease (NCD) targets related to reducing adult obesity and salt intake [1]. Sub-optimal diets, resulting from low consumption of nutritious food and high consumption of harmful foods, are leading risk factors for morbidity and mortality around the world [3, 4]. Additionally, existing food production systems threaten the health of the planet and require urgent transformation [5]. The agriculture sector alone is responsible for 70% of global fresh water use, 23% of greenhouse gas emissions, and significant soil degradation and biodiversity loss [6].

Food system inequalities are being exacerbated by climate change [7]. Low-and middle-income countries have a reduced capacity to adapt to climate change and will face the harshest impacts from low crop yields, high food prices and compromised food utilisation arising from disruptions in household drinking water and increased infectious disease burden [8, 9].

The Gambia, situated on the lower edge of the Sahel, is highly vulnerable to climate change [10]. It experiences irregular rainfall patterns and flooding, together with longer periods of drought, and extreme heat that affect food production and livelihoods [11, 12]. Consequently, domestic food production is lower than national demand for many food items including rice, the national staple, and fruits and vegetables—so food supply is heavily supported through importation from other countries [13, 14]. Ongoing economic and demographic changes such as increased income and high urbanisation rates have also shifted diets away from traditional foods that are high in wholegrain and vegetables towards more processed foods high in refined grains, oils and sugar [15, 16]. These changes have compounded health implications—increasing overweight and obesity and diet related chronic diseases (hypertension and diabetes) in adults [17, 18], and also reduce the resilience of diets by increasing dependence on food trade with associated high water [19] and carbon footprints [20]. Improving the food system to deliver healthy diets could therefore be a means to improve health and increase environmental sustainability [21]. But this will require realignment of the current food systems and consumption patterns—supported by strong political will [22].

Drawing from the best available evidence, the EAT-Lancet Commission in 2019 proposed a universal reference ‘diet for the Anthropocene’ to deliver optimal human health whilst maintaining environmental sustainability [23]. The diet is estimated to sustainably feed the future population and prevent a substantial number of deaths from diet related NCDs [23]. The diet is largely plant-based and emphasises consumption of vegetables, fruits, wholegrains, legumes and nuts, and unsaturated oils. The EAT-Lancet diet recommends low consumption of red and processed meat, added sugar, refined grains and starchy foods. While advocating for a ‘Great Food Transformation’, the Commission recognised that the required changes in diets will have to differ by region and from country to country [23]. Therefore, adoption of the reference diet needs to be carefully tailored to context-specific needs to limit unintended health and environmental impacts [24]. Furthermore, in countries such as The Gambia which has co-existing undernutrition and overnutrition, the required dietary changes might need to vary among different population groups. For example, despite progress over the years in reducing undernutrition, one in five children under five years still suffer from stunting and three in five are anaemic. Increased provision of animal source foods could be important sources of essential nutrients to combat these conditions [25, 26]. At the same time, over 40% of adults are overweight implying excess provision of calories and a need for moderation [27].

Given these problems and existing government efforts to improve on undernutrition [28], diet-related chronic diseases and the environment [29], it is important and timely to examine national dietary patterns and identify major leverage points and opportunities to achieve multiple co-benefits of changing diets among different population groups. Therefore, we assessed deviation of the average Gambian diet from the EAT-Lancet dietary guidelines for healthy and sustainable diets and identified leverage points to simultaneously improve nutritional and planetary health.

## 2. Methods

### 2.1. Study design and survey details

This study analysed the Integrated Household Survey (IHS2015/16) of The Gambia conducted between May 2015 and April 2016 [30, 31], The IHS2015/16 was a comprehensive nationally representative cross-sectional survey with dietary data collected year-round to reflect seasonal changes in diets. The survey was designed to enable comparison of average diets at national and district level and to explore rural-urban differences. It covered data collection on demographic and economic household characteristics. Details of the sampling procedure used by the IHS2015/16 are provided elsewhere [30]. Briefly, the survey used a two-stage probability proportional to size procedure with stratified random sampling. The Gambia Bureau of Statistics defined enumeration areas (EAs) across the eight local government areas (LGAs) (including two municipalities) and districts were selected at the first stage. Each EA was classified as either rural or urban. The next stage involved selection of an equal number of households with equal probability of selection from the household listing in each EA. Overall, 622 EAs and 13 340 households were selected with a response rate of 99.4% (13 281 households interviewed).

### 2.2. Dietary data and processing

The IHS2015/16 collected quantitative household food consumption data using a 7 d recall questionnaire [31]. The questionnaire included 145 food items organised into broad food groups: cereals and products; poultry and products; meat; fish; milk and products; oils and fats; fruits; nuts; starchy roots and tubers; vegetables; sugar, honey and confectionary; and spices and condiments [30]. Our analysis includes consumption of 92 food items excluding spices and condiments (supplementary table S1). Quantities of consumption were reported in both metric units and household measures (e.g. one cup of rice) which were converted into grams of average household intake per person per day by dividing equally by number of people in households. Where household measures were reported without metric unit equivalent estimation, we estimated the gram equivalents using market determined quantities (supplementary material S1).

We calculated total energy intake by linking food intake data with the United Nations Food and Agriculture Organization (FAO) food composition tables for West Africa [32]. The US Department of Agriculture’s food composition data [33] and Gambia specific tables [34] were used where food items were not contained in the West African tables.

### 2.3. Covariates

Most covariates were at household level unless specified otherwise. The covariates considered for multivariable regression modelling were: household wealth; remittances per capita; seasonality; crop production diversity; total energy; sex of household head; ethnicity; education of household head; household size; and urbanisation status (supplementary material S2).

### 2.4. The sustainable and healthy diet index (SHDI) score

The EAT-Lancet reference diet corresponds to 2500 kcal per day energy needs for a 30-year-old woman weighing 60 kg with moderate to high physical activity level. It sets serving averages and suitable ranges (in grams per person per day) for each food group to reflect diets associated with greater health and environmental sustainability [23]. To measure adherence to, or deviations from, the proposed diet, we developed an EAT-Lancet diet index (the ‘sustainable and healthy diet index (SHDI)’) by combining two previous scoring methods [35, 36] (Method 1 and Method 2 respectively) to reflect The Gambian food system, nutritional needs and eating patterns. Method 1 scores consumption of food groups to reflect on micronutrient adequacy [35]. The method assigns one point for intakes within the EAT-Lancet range for each food group and zero points for consumption outside of the range. The method does not assign positive scores for zero intakes of essential food groups. The ranges of intake recommended by EAT-Lancet often include zero intake values (e.g. 0–14 grams for beef and lamb and 0–14 grams for pork) to allow for interchangeability and replacement between closely related food groups [23]. However, in many food insecure or minimally food secure areas, such as The Gambia, there is low availability of alternatives to replace non-consumed food groups. Consequently, assigning positive scores for non-consumption of these foods could be a proxy for inadequate intake of micronutrients [35]. In these instances, the mean of the target range is taken as the lower bound instead of zero [35]. We adopted this approach by Method 1 to ensure greater micronutrient adequacy of the resulting index.

The scoring approach used by Method 2 reflects risk of NCD and associated mortality [36] by assigning scores 0–3 depending on different levels of consumption of food groups. The method assigns higher scores for greater consumption of ‘emphasised’ food groups for which high consumption is good for health (vegetables, fruits, unsaturated oils, legumes, nuts, wholegrains, and fish) while points are taken away for higher consumption of ‘limited’ food groups for which overconsumption is bad for health (beef and lamb, pork, poultry, eggs, dairy, potatoes, and added sugar) [36]. Method 2 does not make micronutrient adequacy considerations for nutrient-rich food groups including meat and dairy which may lead to diets rich in these food groups being penalised under this scoring system.

In our combined EAT-Lancet index (SHDI), we used the points distribution system similar to Method 2 (0–3 points) and applied micronutrient adequacy considerations for nutrient-rich food groups similar to Method 1. Therefore, in food groups where the EAT-Lancet range includes zero intake as the lower bound such as beef and lamb (0–14 grams) we used the mean of the target range to represent the lower bound instead of zero (i.e. 7–14grams) (Method 1). We then assigned the scores (0–3 points) according to consumption within or outside this new range avoiding positive points for zero intake (table 1 and supplementary material S3). This avoids assigning a positive score for non-consumption of these food groups as would result from applying Method 2.

**Table 1.** Scoring system used to generate the SHDI.

EAT-Lancet food group	EAT-Lancet target intake (range in grams d^−1^)	Intake range with minimum intake values (g d^−1^)^a^	Score^b^			
			3	2	1	0
All vegetables	300 (200–600)	200–600	>300	200–300	100–200	<100
All fruits	200 (100–300)	100–300	>200	100–200	50–100	<50
Unsaturated oils	40 (20–80)	20–80	>40	20–40	10–20	<10
Beans, lentils and peas^c^	75 (0–150)	75–150	>75	37.5–75	18.75–37.5	<18.75
Peanuts and tree nuts	50 (0–100)	50–100	>50	25–50	12.5–25	<12.5
Wholegrains^c^	116 (0–232)	116–232	>116	58–116	29–58	<29
Potatoes and cassava	50 (0–100)	50–100	>50	25–50	12.5–25	<12.5
Fish	28 (0–100)	28–100	>28	14–28	7–14	<7
Palm oil	6.8 (0–6.8)	⩽6.8	<1.7	1.7–3.4	3.4–6.8	>6.8
Added sugar	31 (0–31)	15.5–31	<7.75	7.75–15.5	15.5–31	>31
Refined grains^c^	116 (0–232)	116–232	<116	58–116	116–232	>232
Beef and lamb	7 (0–14)	7–14	7–14	3.5–7	1.75–3.5	<1.75 or >14
Pork	7 (0–14)	7–14	7–14	3.5–7	1.75–3.5	<1.75 or >14
Poultry	29 (0–58)	29–58	29–58	14.5–29	7.25–14.5	<7.25 or >58
Dairy	250 (0–500)	250–500	250–500	125–250	62.5–125	<62.5 or >500
Eggs	13 (0–25)	13–25	13–25	6.5–13	3.25–6.5	<3.25 or >25

^a^ Based on Method 1 [35] except for refined grains and palm oil where we allowed positive scoring for non-consumption. Our approach treats beef & lamb and pork as two groups and splits grains into wholegrains and refined grains.

^b^ Scores were assigned based on Method 2 [36] with exceptions to: wholegrains, beef and lamb, pork, poultry, dairy, and potatoes and cassava where we used different criteria and avoided awarding points for non-consumption. We awarded points differently for added sugar intake by taking points away for intakes above the upper limit while Method 2 assigned positive scores for intakes up to 200% of the upper limit.

^c^ Grains (whole and refined), beans, lentils and peas are dry, raw and includes soy foods consistent with EAT-Lancet recommendations. EAT-Lancet recommendations for grains are combined with target 232 g (0–464 g). These were split in this report to reflect local availability and consumption patterns.

Overall, 16 food groups (based on EAT-Lancet recommendations with some modifications) were defined in this study with each having a maximum score of three points depending on intake level—resulting in a total maximum score of 48 for the composite SHDI. Further details about the scoring criteria used for each food group and specific modifications applied to the SHDI are shown in table 1 and supplementary material S3.

### 2.5. Statistical analyses

Scores obtained for each food group were summed to form a composite SHDI. The proportion of total index scores over the total expected scores was used as an indicator of the level of adherence to the EAT-Lancet recommendations. We used both graphical methods and descriptive statistics to explore and summarise the data and to elicit patterns.

We fitted multivariable mixed effects regression models specifying ‘region’ as a random effect and other covariates as fixed effects using maximum-likelihood estimation to assess factors associated with a 1-unit increase or decrease in the SHDI. Potential multicollinearity among variables was assessed using the variance inflation factor by including the variables of the fully adjusted model in a normal least squares regression. All statistical analyses were performed using Stata version 16.1 (StataCorp, USA).

## 3. Results

From an initial sample of 13 281, the current analysis includes 12 713 households after data processing to exclude households reporting extreme energy intakes (supplementary table S2). Proportional to the population distribution, the majority of the households in the sample were based in the Central River region (24.5%) and the West Coast region (22.2%), with the smallest number of households reporting from Banjul (5.6%). More than two thirds of the households were in rural areas (75.6%), 84.8% were headed by a male family member, and 76.1 of the households had a head without schooling. More than seven in ten households reported to grow at least one crop (77.4%) and approximately two in five households received remittances during the last year (37.7%) (supplementary table S3).

### 3.1. Mean consumption of energy and food groups

Average daily energy intake per capita was 2536 kcal with wide regional variation. The lowest energy intake was recorded in West Coast region (2011.0 kcal day^−1^) while those in Central River and Upper River regions had the highest energy intake of 2912.9 kcal per person per day compared to the 2500 kcal person day^−1^ recommended by EAT-Lancet. Households in rural areas (2616.9 kcal person day^−1^) consumed more energy than their counterparts in urban areas (2284.3 kcal person day^−1^). There was 100 kcal more energy intake on average in the rainy season than in the dry season (2512.6 kcal person day^−1^). Households headed by men also had higher energy intakes over female headed households (table 2 and supplementary table S4). Consumption of different food groups differed by household and geographic characteristics. For example, consumption of fruit and vegetables was high among household members in urban areas compared to rural areas, but households in urban areas also consumed relatively higher amounts of sugar, and beef and lamb. In contrast, total grain intake was higher among rural households than urban households. Furthermore, the intake of fruits, vegetables, and grains (whole and refined) varied more by season than other food groups. While fruit and vegetable consumption was higher in the dry season compared to the rainy season, the reverse was true for consumption of grains (table 2 and supplementary table S4).

**Table 2.** Background characteristics of sampled households, energy, and food group consumption.

EAT-Lancet food group	Percent of households consuming each food group per day (national) % (95% CI)	Mean consumption of food groups (national) g day^−1^	Type of settlement mean (95% CI) g day^−1^		Season mean (95% CI) g day^−1^		Household head mean (95% CI) g day^−1^	
			Urban	Rural	Rainy	Dry	Male	Female
Total energy		2536.2 (2514.3–2558.2)	2284.3 (2247.3–2321.2)	2616.9 (2590.6–2643.2)	2616.1 (2568.4–2663.9)	2512.6 (2487.9–2537.3)	2545.6 (2521.4–2569.8)	2483.6 (2431.7–2535.5)
All vegetables	96.6 (96.0–97.0)	153.9 (151.5–156.3)	206.2 (200.7–211.7)	137.2 (134.7–139.7)	145.6 (140.7–150.6)	156.4 (153.7–159.1)	144.4 (141.9–146.8)	207.4 (200.2–214.5)
All fruits	37.2 (36.3–38.0)	53.1 (50.7–55.6)	77.8 (72.0–83.6)	45.2 (42.7–47.7)	22.6 (20.3–24.9)	62.1 (59.2–65.1)	50.9 (48.4–53.4)	65.3 (58.3–72.4)
Unsaturated oils	87.2 (86.6–87.7)	21.8 (21.4–22.1)	24.0 (23.2–24.7)	21.1 (20.7 (21.4)	21.6 (20.9–22.4)	21.8 (21.4–22.2)	20.9 (20.6–21.3)	26.6 (25.6–27.6)
Beans, lentils and peas	26.5 (25.7–27.2)	8.6 (8.2–9.0)	5.1 (4.5–5.6)	9.7 (9.3–10.2)	8.8 (8.0–9.6)	8.5 (8.1–8.9)	8.9 (8.5–9.3)	6.7 (5.8–7.6)
Peanuts and tree nuts	67.3 (66.5–68.1)	19.1 (18.4–19.7)	15.3 (14.1–16.6)	20.2 (19.5–20.9)	18.9 (17.5–20.3)	19.1 (18.4–19.8)	19.2 (18.6–19.9)	17.9 (16.3–19.5)
Wholegrain	19.8 (19.1–20.5)	41.7 (39.7–43.8)	6.7 (5.1–8.2)	53.0 (50.3–55.6)	51.1 (46.4–55.9)	39.0 (36.7–41.3)	47.0 (44.6–49.4)	12.4 (9.6–15.2)
Potatoes and cassava	48.4 (47.5–49.5)	18.8 (18.3–19.4)	34.9 (33.3–36.4)	13.7 (13.2–14.3)	18.0 (16.8–19.3)	19.1 (18.4–19.7)	17.6 (17.0–18.2)	25.5 (23.8–27.3)
Fish	93.8 (93.4–94.2)	81.1 (79.9–82.3)	93.5 (90.7–96.4)	77.1 (75.8–78.5)	79.9 (77.3–82.6)	81.4 (80.0–82.8)	76.3 (75.1–77.6)	107.8 (103.9–111.6)
Palm oil	68.8 (68.0–69.6)	7.6 (7.4–7.7)	9.1 (8.7–9.4)	7.1 (6.9–7.3)	8.1 (7.7–8.4)	7.4 (7.3–7.6)	7.2 (7.1–7.4)	9.6 (9.1–10.1)
Added sugar	96.4 (96.1–96.7)	66.5 (65.6–67.5)	76.7 (74.4–79.0)	63.3 (62.3–64.3)	65.4 (63.4–67.4)	66.9 (65.8–67.9)	65.8 (64.8–66.8)	70.7 (68.2–73.2)
Refined grains	98.8 (98.6–99.0)	411.6 (407.0–416.1)	352.5 (345.8–359.3)	430.4 (424.9–436.0)	429.6 (419.9–439.4)	406.2 (401.1–411.3)	415.7 (410.7–420.8)	388.1 (378.3–397.9)
Beef and lamb	28.4 (27.7–29.2)	12.4 (11.8–12.9)	17.5 (16.1–18.8)	10.7 (10.2–11.2)	12.5 (11.4–13.6)	12.3 (11.7–12.9)	12.2 (11.6–12.8)	13.2 (11.9–14.5)
Poultry	26.8 (26.0–27.6)	12.3 (11.8–12.8)	18.6 (17.2–19.9)	10.3 (9.8–10.8)	12.3 (11.3–13.3)	12.3 (11.7–12.9)	11.5 (11.0–12.0)	17.0 (15.3–18.7)
Dairy	44.0 (43.2–44.9)	26.6 (25.5–27.7)	21.8 (20.0–23.6)	28.1 (26.8–29.4)	27.6 (25.3–29.9)	26.3 (25.0–27.5)	27.1 (25.9–28.4)	23.5 (20.9–26.0)
Eggs	13.8 (13.2–14.4)	1.3 (1.2–1.4)	3.2 (2.9–3.5)	0.7 (0.6–0.8)	1.4 (1.2–1.6)	1.3 (1.2–1.4	1.2 (1.1–1.3)	1.6 (1.4–1.9)

Pork is excluded from table as consumption ⩽0.5 g day^−1^. CI: Confidence Interval

### 3.2. Household food consumption and adherence to EAT-Lancet diet recommendations

**Figure 1.**
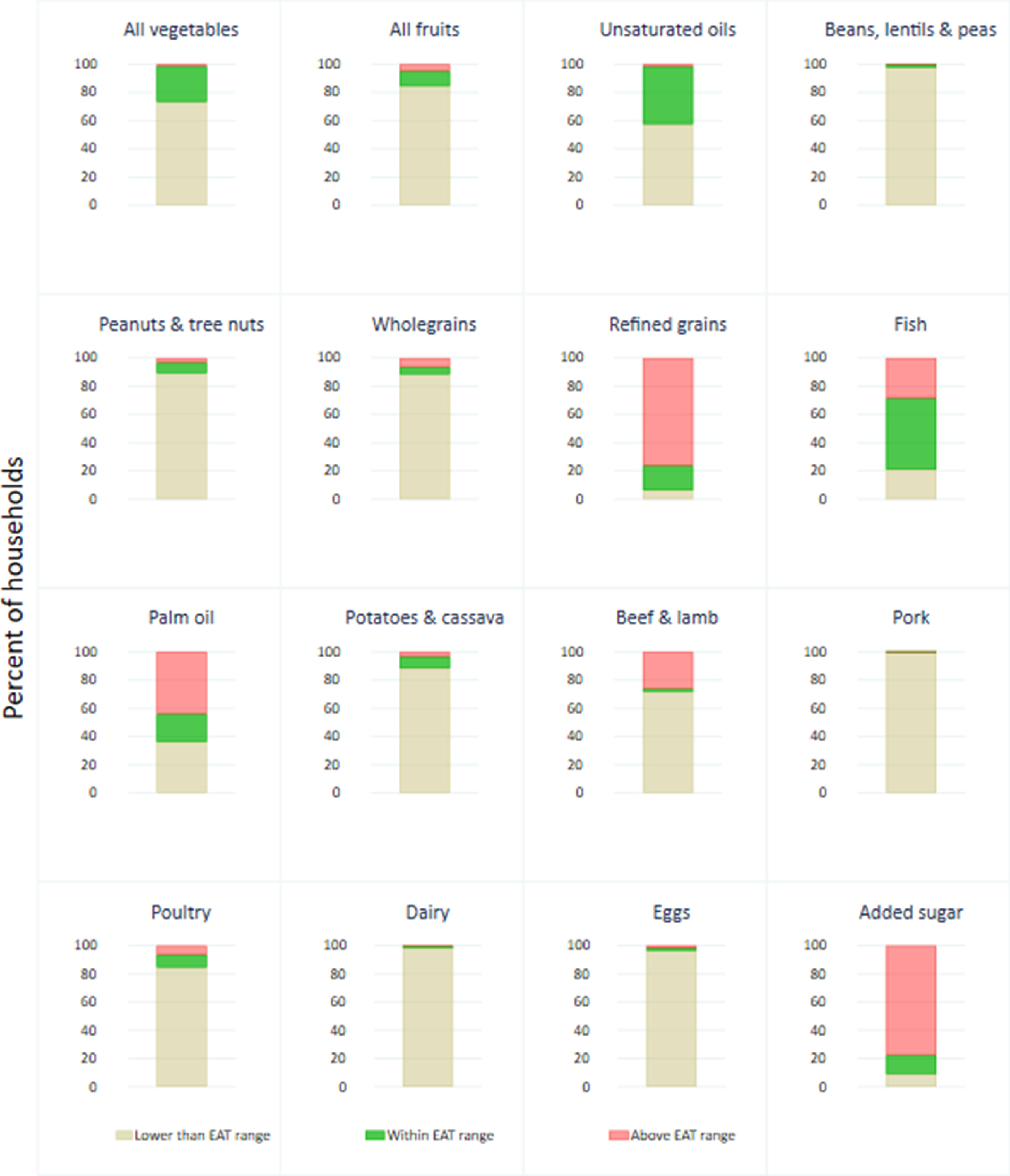
Comparison of mean household food group consumption with EAT-Lancet ranges [Recommended ranges are according to Method 1 with minimum values as described in the ‘intake range’ column of table 1].

Comparing mean intakes to the EAT-Lancet targets, only a small proportion of household members had intakes falling within the recommended range (figure 1). Food groups with a relatively high proportion of households consuming within the EAT-Lancet target include: fish (50.5%) and unsaturated oils (40.8%)—all other food groups had a quarter or less of households with consumption falling within the EAT-Lancet range. The majority of household members had mean intakes above the upper limit recommended by EAT-Lancet for added sugar (77.5%), refined grains (76.1%), and palm oil (43.9%). For all other food groups (fruits, vegetables, pork, beef and lamb, eggs, dairy, poultry, potatoes and cassava, wholegrains and peanuts and tree nuts), most households consumed below the lower range (figure 1). However, more than 20% of households were also consuming above the upper range for beef and lamb and fish. Consumption above the recommendation for beef and lamb and fish was higher among the wealthiest households and those residing in urban areas (supplementary table S5).

**Figure 2.**
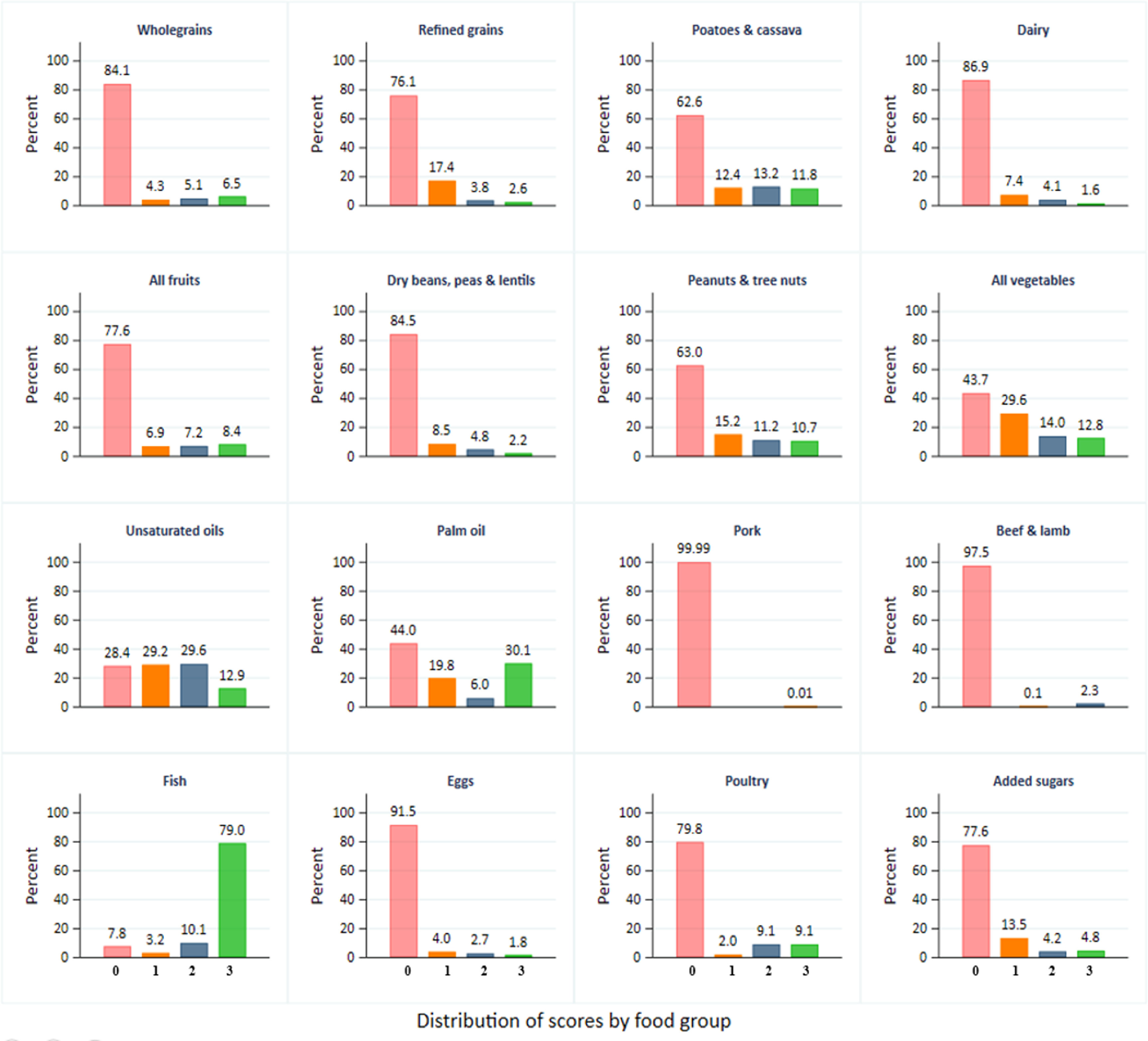
Distribution of scores obtained on each of the 16 food groups [Scores 1, 2 and 3 generally refer to consumption within acceptable limits of a food group. A 0 score generally means consumption outside acceptable limits for a food group (details in table 1). Scores 0, 1, 2 & 3 are shown by light shades of red, orange, blue and green respectively. Scores with 0% households are not shown].

Consequently, the overall score on the SHDI was very low, with a mean composite index of 10.1/48 (SD = 3.7) and a highest reported score of 28.0/48 (supplementary figure S1). The distribution of SHDI scores obtained by households for each food group is presented in figure 2. Note that failure to meet targets for any individual category does not imply an overall failure because substitutions are permitted.

**Figure 3.**
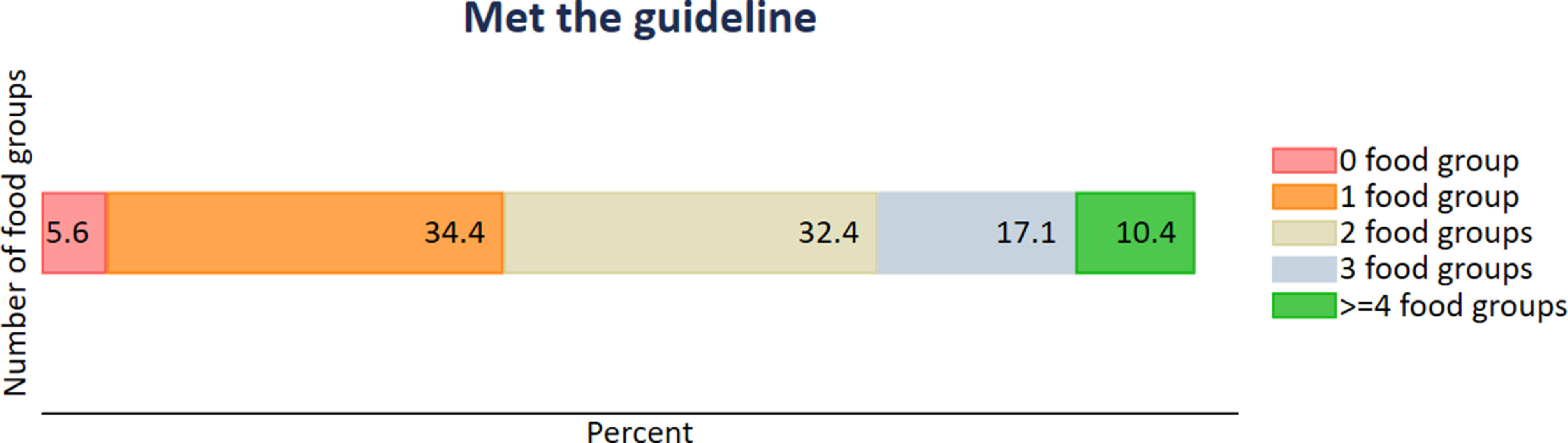
Percent of households meeting the EAT-Lancet guidelines by number of food groups [Total number of food groups = 16. Meeting guidelines here is defined by obtaining a score of 3 in each food group].

Considering adherence by individual food groups and consistent with figure 1, most households (79%) scored three points for fish intake. The proportion of households scoring three points for other food groups was lower than 50%. For refined grains, most households (76%) scored zero points due to over consumption, but most households (84%) scored zero points on wholegrain intake due to extreme under consumption. Many households (>70%) also reported very low or no consumption of fruits, beans, lentils and peas, beef and lamb, dairy, pork, eggs, and chicken, resulting in scoring zero points in these food groups (figure 2). Overall, 66.8% of households scored three points in one or two food groups (comprising mainly fish and palm oil) and only 10% scored three points in four or more food groups (these were mainly fish, palm oil, vegetables, and unsaturated oils) (figure 3 and supplementary table S6). However, households that scored three points on only one food group also scored two points on a number of other food groups for which consumption did not reach a three score (supplementary table S6).

### 3.3. Determinants of increased sustainable and healthy diet index (SHDI) score

The adjusted mixed effects model (accounting for per capita energy intake and receipt of remittances) showed that diets of female headed households scored 0.32 points higher on the SHDI than diets of male headed households (95% CI: 0.14–0.50, *P* < 0.001). Similarly, diets of households in urban areas scored 0.61 points higher compared to those in rural areas (95% CI: 0.41–0.81, *P* < 0.001). There was a positive association between household wealth index and the SHDI (*β* = 0.37; 95% CI: 0.32–0.43, *P* < 0.001). Furthermore, in households where the head had no schooling, there was a lower mean dietary index score (*β* = −0.41; 95% CI: −0.57-(−0.26), *P* < 0.001) compared to households where the head received some schooling. There was evidence of a strong relationship between ethnic group and dietary index score: compared to Mandinkas, other ethnicities (Fula, Jola and Serahulleh) showed lower index scores, except the Wollof who scored higher (table 3). Diets in the dry season scored on average 0.47 points higher on the index than those in the rainy season (95% CI: 0.32–0.61, *P* < 0.001). Crop production diversity was also positively associated with the SHDI (*β* = 0.14; 95% CI: 0.10–0.19, *P* < 0.001) while each additional family member was associated with a marginal decrease in dietary score of 0.02 points (−0.04-(−0.01), *P* < 0.001) (table 3).

**Table 3.** Mixed effects regression analysis of the determinants of EAT-Lancet diet index in The Gambia.

	EAT-Lancet index score (*β*) (95% confidence interval)^a^	P-value
Household head		<0.001
Male	0 (base)	
Female	0.32 (0.14–0.50)	
Area of residence		<0.001
Rural	0 (base)	
Urban	0.61 (0.42–0.81)	
Wealth quintile	0.37 (0.32–0.43)	<0.001
Ethnicity/tribe		<0.001
Mandinka/Jahanka	0 (base)	
Fula/Tubular/Lorobo	−0.31 (−0.47− (−0.15))	
Wolof	0.37 (0.17–0.58)	
Jola/Karoninka	−0.02 (−0.29–0.25)	
Serahulleh	−0.01 (−0.30–0.28)	
Other	−0.07 (−0.36–0.22)	
Household head ever attended school		<0.001
Yes	0 (base)	
No	−0.41 (−0.57− (−0.26))	
Season		<0.001
Rainy	0 (base)	
Dry	0.48 (0.32–0.61)	
Crop diversity score	0.14 (0.10–0.19)	<0.001
Remittances (per capita)	9.17 × 10^−6^ (1.58 × 10^−6^−1.67 × 10^−5^)^b^	0.02
Household size	−0.02 (−0.04-(−0.01))	0.001
Total energy	9.24 × 10^−4^ (8.71 × 10^−4^−9.77 × 10^−4^)^b^	<0.001

^a^ Coefficients are adjusted for all other variables in the model.

^b^ e = x10 exponentiation.

## 4. Discussion

### 4.1. Main findings

In this study, we have demonstrated that average Gambian diets typically show strong deviations from the healthy and sustainable dietary guidelines as proposed by the EAT-Lancet Commission. The diet was dominated by consumption of less-healthy options such as refined grains and added sugars which exceeded the recommendations, whilst intake of nutritionally important food groups such as fruits, vegetables, dairy, and poultry were much lower than the EAT-Lancet targets. Less than a third of the population consumed beef and lamb, with many non- or very low consumers with low wealth indices and living in rural areas while there were some over-consumers of these foods in the wealthiest and urban households. Relatively high meat intake among urban dwellers is consistent with the wider sub-Saharan Africa region [37]. Importantly, we show that, consistent with similar low-and middle-income settings [38], the diet does not fulfil nutritional requirements but consumption is often within recommended levels for food components known to impact heavily on the environment (such as livestock products) [21]. We also identified important socioeconomic characteristics that could serve as leverage points for improving healthiness whilst not impacting on the sustainability of diets in The Gambia.

### 4.2. Research in context

Diets in The Gambia typically show low dietary diversity [39], which partly explains the low levels of alignment with the healthy and sustainable dietary guidelines by the EAT-Lancet Commission. A less diverse diet implies that a few food groups are over consumed (e.g. polished white rice, bread, oils, and added sugar) while others are under consumed; both cases result in deviations from the EAT-Lancet targets.

The higher scores on the SHDI in the dry season compared to the rainy season are consistent with results from Kenya and Vietnam [35]. As other food groups remained relatively unchanged throughout the year, higher availability of fruit and vegetables during the dry season as compared to the rainy season explains much of the difference in scores. High SHDI scores among those with better wealth, schooling and urban settlement also seem to be driven by availability and consumption of fruits and vegetables. Furthermore, female-headed households have been shown by past studies to spend a higher proportion of income towards household food and nutrition (in this case, on more vegetables) than males [40]. This underscores the overarching importance of fruit and vegetables in improving diets and meeting healthy and sustainable diet targets in The Gambia. Previous evidence shows that increased production diversity is associated with diet diversity [41], which may explain the positive association between SHDI and home agricultural production found in this study. Contrary to a recent meta-regression that found higher vegetables intake in rural areas as compared to urban areas [37], urban residence could be a good proxy for better access to food markets and income than in rural areas and may explain the higher SHDI scores associated with urban settlement in this study [42]. Finally, while remittances are a key source of household income in The Gambia [43] and linked with improvement in food and nutrition security in sub-Saharan Africa [44], they were only weakly associated with the sustainable diet index in this study. This could be due to using income from remittances to buy less healthy food groups (such as refined grains and sugar) or for purchases of non-food household items.

### 4.3. Implications including policy recommendations

If The Gambia was to successfully promote healthy and sustainable dietary guidelines, such as those proposed by the EAT-Lancet commission, it would require substantial shifts in current food supply and consumption patterns. Chiefly, it would involve increasing the supply and intake of fruits, vegetables and wholegrains as well as ensuring that they are available in all parts of the country throughout the year. This would have to go along with cutting down on refined grains (polished white rice and bread) and added sugar. Also, the current low amounts of livestock products with a dominant aquatic source of protein (mainly fish) would need to be maintained to remain within health and sustainability limits. The national average intake of dairy products lies far below the 250 g EAT-Lancet target and needs to increase to improve the nutrient content of the diet. These changes would involve a careful consideration of the food supply and demand side dynamics. Improving food choice and demand can drive food supply under favorable structural factors that enable adequate supply from both domestic production and import sources [45]. However, a variety of other factors that influence food choice would need to be tackled—including the affordability of food groups that are currently under-consumed, nutrition education about the importance of dietary diversity, food preferences, and marketing practices [46].

In many low- and middle income countries, one of the main barriers to consuming the recommended amount of fruit and vegetables as per the EAT-Lancet recommendations is their affordability [47]. In addition to high cost, the supply of fruit and vegetables from both domestic production and imports from other countries is often insufficient: in the Gambia, average per capita fruit and vegetable supply falls short of national demand [48, 49], and would need to be doubled/tripled (especially for fruits) to meet the EAT-Lancet recommendations. This trend is noticeable in other low- and middle-income settings. For example, in India, an additional US $1.0 was required per household member per day in order to purchase the amount of fruit and vegetables (as well as other food groups) recommended in the EAT-Lancet diet [50]. High price volatility of fruit and vegetables in different seasons further complicated the affordability question [50].

Given the clear discrepancy between current supply and fruit and vegetable supply required for population-wide shifts to healthy and sustainable dietary guidelines in The Gambia, adequate action to improve supply streams would be crucial. This could involve various pathways including investments by government and development partners to increase the supply of fruits and vegetables, but also methods to overcome seasonal variability in supply, such as preservation methods and introduction of early and late cropping varieties.

The Gambia relies heavily on imported refined grains as the major staple foods [51] likely due to their relative low prices, high convenience for cooking and eating, and prestige associated especially with consumption of rice compared to alternative grains [52]. For alternative and more healthy wholegrains to gain dominance over refined rice in The Gambia, the factors that make rice attractive will need to be equalised. Promoting wholegrain alternatives such as pearl millet and maize to reduce reliance on rice and refined wheat bread could have multiple benefits: higher consumption of wholegrains would likely impact positively on both health and the environment. For instance, millet production is associated with lower greenhouse gas emissions compared to rice [53] and it can easily be eaten as wholegrain compared to rice that is almost always refined. In addition, millets are relatively more adaptable to the local climate than rice as millet has been grown traditionally over decades in The Gambia [54]. There are further advantages for promoting millet as an alternative to rice because it is already the second most consumed grain in the country (especially in rural areas) and has multiple local recipes [51]. The low consumption of millet is largely the result of inadequate supply and low convenience in its processing and preparation which limits uptake especially among busy urban dwellers—this needs to be addressed to enable nationwide scale-up.

Locally sourced fish is the main source of animal protein in the diet together with low amounts of poultry and livestock products in The Gambia. Although this combination is often estimated to ensure environmental sustainability [21], possible overexploitation of local fish stocks by foreign fish-meal factories [55] greatly threatens the sustainability and long term resilience of fish supply for the future [56]. This is particularly relevant as fish is often the only nutrient-rich food consumed in adequate amounts by those with less diverse diets. Hence a declining supply (and potential price increase) would make fish inaccessible and/or unaffordable in this group, which could disproportionally disadvantage them—reducing diet quality.

Currently average beef and lamb consumption is low or zero for the majority of the population, while a smaller proportion of (mostly) urban dwellers have higher intake than recommended by the EAT-Lancet diet. In the Global North red meat is grossly overconsumed by many and serves as a major source of food system emissions—the relatively small amounts of red meat consumption as recommended by sustainable and healthy dietary guidelines often mean a substantial cut in meat consumption for the majority of people in the Global North if they wish to adhere to such guidelines. In The Gambia, this is however more complex: given the double burden of malnutrition, low dietary diversity and the relatively low environmental impact of agriculture (including that of livestock—kept extensively and fed mainly on low-opportunity cost biomass such as grass with lower environmental impact compared to those kept intensively and fed with cereals [57]), blanket meat reduction strategies are less of a useful option to improve healthfulness and sustainability in the immediate term. In fact, for the majority of the Gambian (rural) population the small amount of red meat as recommended in healthy and sustainable dietary guidelines would in fact translate into an increase in red meat consumption as compared to their current diets. Therefore, a more targeted approach to nutrition education is required to facilitate changes in red meat consumption patterns across different population groups that are appropriate to need.

Cutting down on the amount of added sugar in the Gambian diet will improve diet quality. Until 2011, the Gambian government provided tax waivers on imported sugar [58]. In spite of the removal of tax exemptions, Food Balance Sheet data from the FAO show that daily sugar supply in Gambia has continued to increase (from 83 g person day^−1^ in 2011 to 99 g person day^−1^ in 2019) [59]. This may imply that sugar is still cheaper than the minimum threshold required to reduce demand. Possible strategies to reduce sugar consumption may involve a combination of approaches with complementary effects including taxation, food based dietary guidelines and social behaviour change communication.

### 4.4. Strengths and limitations

This study has several strengths. First, we used a national sample with multiple variables which allowed examination of diets at sub-national and household levels. The design of the survey also allowed us to assess diets in different seasons of the year for a more comprehensive understanding of the national diet. Our scoring system has also allowed a good understanding of the extent of deviation and conformity of different food groups to the EAT-Lancet diet targets and to identify leverage points for improvement.

However, the study also has several limitations. We estimated an average household intake per person and compared this to a reference diet. This did not allow us to explore potential non-equitable food distribution among household members (for example, children may be consuming less energy overall but they may also eat more seasonal fruits than adults) [60]. There was also a possibility for bias by comparing average intakes to a standard reference diet. For example, rural and more farming dominated households may be more physically active than urban dwellers, by comparing to the standard reference diet this likely introduced a bias in which overconsumption by urban dwellers is underestimated and underconsumption by rural dwellers is underestimated. The 7 d food frequency questionnaire method of dietary intake assessment is vulnerable to recall problems but is more likely to reflect the ‘regular diet pattern’ of households than shorter recall periods such as 24 h recalls. Additionally, the EAT-Lancet diet targets are more focused on adults [23] and may not apply directly to nutritionally vulnerable groups such as young children or pregnant and lactating women [61]. Furthermore, the EAT-Lancet diet is more plant-based, implying a dominant plant-source iron supply which is less bioavailable as compared to haem-based sources [62]. Therefore, for the diet to provide optimal iron nutrition and reduce existing high levels of iron deficiency and anaemia in the population [61], promotion of the EAT-Lancet diet in this setting needs to particularly emphasise adequate supply and consumption of appropriate amounts of livestock products (rich source of bioavailable iron and also enhance absorption of plant source iron) as well as fruit and vegetables (high in vitamin C to aid absorption of plant-source iron [63]). Our assessment of an average diet limited our ability to identify specific food group combinations that may exist within population sub-groups that are often identified through dietary pattern analysis [64]. However, the use of average diets of population groups (by region, settlement type etc) as done in this study may also be useful in relatively smaller populations such as The Gambia with more homogeneity in food supply and consumption [65].

## 5. Conclusion

We can conclude that the current Gambian diet is high in less healthy food groups such as refined grains and added sugar and is low in nutritionally important food groups such as fruits and vegetables, and average diets currently do not map well onto sustainable and healthy dietary guidelines. Opportunities to improve on the healthiness of diets, while potentially increasing sustainability and resilience, could be found by focussing on the substitution of refined grains by wholegrains, reducing added sugar consumption and year-round supply of fruit and vegetables.

## Data Availability

The Integrated Household Survey 2015/16 data analysed in this study are publicly available from the World Bank (https://microdata.worldbank.org/index.php/catalog/3323).
